# Pharmaceutical Analysis Model Robustness From Bagging-PLS and PLS Using Systematic Tracking Mapping

**DOI:** 10.3389/fchem.2018.00262

**Published:** 2018-07-06

**Authors:** Na Zhao, Lijuan Ma, Xingguo Huang, Xiaona Liu, Yanjiang Qiao, Zhisheng Wu

**Affiliations:** ^1^Key Laboratory of Xinjiang Phytomedicine Resources and Utilization, Ministry of Education, School of Pharmacy, Shihezi University, Shihezi, China; ^2^Beijing University of Chinese Medicine, Beijing, China; ^3^Pharmaceutical Engineering and New Drug Development of TCM of Ministry of Education, Beijing, China; ^4^School of Integrated Traditional Chinese and Western Medicine, Binzhou Medical University, Yantai, China

**Keywords:** multivariate calibration, near infrared spectroscopy, processing trajectory, Bagging-PLS, variable selection

## Abstract

Our work proved that processing trajectory could effectively obtain a more reliable and robust quantitative model compared with the step-by-step optimization method. The use of systematic tracking was investigated as a tool to optimize modeling parameters including calibration method, spectral pretreatment and variable selection latent factors. The variable was selected by interval partial least-squares (iPLS), backward interval partial least-square (BiPLS) and synergy interval partial least-squares (SiPLS). The models were established by Partial least squares (PLS) and Bagging-PLS. The model performance was assessed by using the root mean square errors of validation (RMSEP) and the ratio of standard error of prediction to standard deviation (RPD). The proposed procedure was used to develop the models for near infrared (NIR) datasets of active pharmaceutical ingredients in tablets and chlorogenic acid of *Lonicera japonica* solution in ethanol precipitation process. The results demonstrated the processing trajectory has great advantages and feasibility in the development and optimization of multivariate calibration models as well as the effectiveness of bagging model and variable selection to improve prediction accuracy and robustness.

## Introduction

Multivariate calibration is the process of relating the measured response to the analyte amounts, concentrations, or other measured values of physical or chemical properties. Partial least squares (PLS) regression is the most effective and commonly used regression techniques in multivariate calibration because of its calibration model quality and ease of implementation. The statistical results show that approximately 20,000 published papers reports used PLS models from 2005 to 2017. The PLS technique has been effectively applied to different fields, especially in pharmaceutical analysis.

Kachrimanis et al. developed a fast and precise method using FT-Raman spectroscopy alongside with PLS for the quantitation of monoclinic and orthorhombic paracetamol in powder mixtures (Kachrimanis et al., 2007). Yu et al. established a PLS model using near infrared spectroscopy (NIR) and gas chromatography data to determine *l*-borneol in Blumea balsamifera (Ai-na-xiang) samples (Yu et al., 2017). Sarkhosh et al. developed a PLS model of redox potential with genetic algorithms selecting pixels in multivariate image analysis for a quantitative structure-activity relationships (QSAR) study of trypanocidal activity for quinone compounds (Sarkhosh et al., 2014). Üstün et al. built a fast quantification method combining ^1^H NMR spectroscopy with PLS to determine the chondroitin sulfate and dermatan sulfate in danaparoid sodium (Üstün et al., 2011). Wu et al. used NIR as a process analytical technology and developed the PLS model of 11 amino acids to monitor their concentration change during hydrolysis process of Cornu Bubali (Wu et al., 2013b).

The successful application of PLS depends on the development and validation of multivariable models. Recently, the multivariate data needs a more suitable method to establish a robust and reliable PLS model. However, many parameters need to be optimized for a quantitative PLS model, which include spectral pretreatment, variable selection, calibration methods, etc. To improve model performance, the pretreatments are used to reduce the undesirable variations effects from instrument, environment, sample preparation protocol, etc. (Faber, 1999; Blanco et al., 2007; Fernández-Cabanás et al., 2007; Lim et al., 2016).

Besides, variable selection in modeling is also an important step to identify informative features and/or remove uninformative variables for better prediction performance and model complexity reduction. Recently, based on the PLS algorithm, some variable selection methods have been developed including interval partial least-squares (iPLS) (Saudland et al., 2000), backward interval partial least-square (BiPLS) (Leardi and Nørgaard, 2004) and synergy interval partial least-squares (SiPLS) (Munck et al., 2001), etc. Many studies have confirmed the efficiency of these variable selection methods for improving model performance (Chen et al., 2008; Di et al., 2010; Wu et al., 2013a; Mahanty et al., 2016).

In addition, a single model is often not robust because of the change of calibration data and model parameters. An alternative effective approach to improve model robustness is ensemble modeling that establishes multiple models and combines their predictions into a single value. Bagging-PLS is one of most important ensemble modeling techniques. About 60 papers were published on the use of Bagging-PLS model in the period 2005–2017. Galvão et al. used bagging strategies in conjunction with Multiple Linear Regression (MLR) and PLS to develop the multivariate calibration models for four diesel quality parameters, showing that the prediction accuracy was improved by subagging procedure (Galvão et al., 2006). Pan et al. combined ensemble method of Bagging with PLS to detect naringin, hesperidin and neohesperidin in pilot-scale extraction process of *Fructus aurantii* with online NIR sensors (Pan et al., 2015).

Most of the published works dealing with PLS model used a univariate to optimize these modeling parameters step by step according to the root-mean-square error. The number of modeling paths of this method was limited and the results were often not the global optimal. Then, we proposed processing trajectory that can provide a systematic way to optimize parameters in a quantitative model (Zhao et al., 2015).

Based on the above considerations, we extend the optimization of spectral pretreatment, latent factors and variable selection using tracking procedure to spectral pretreatment, latent factors, variable selection and calibration method. The methods of variable selection included iPLS, BiPLS, and SiPLS. The models were established by using PLS and Bagging-PLS. The model performance was assessed using the root mean square errors of validation (RMSEP) and the ratio of standard error of prediction to standard deviation (RPD) (Esbensen et al., 2014; Williams et al., 2014). Two diferent NIR spectral datasets (one standard and one open source) were analyzed. The proposed procedure was used to predict active pharmaceutical ingredients (API) in tablets and chlorogenic acid of *Lonicera japonica* solution in ethanol precipitation process.

## Datasets and analysis

### Datasets

#### Tablet

The NIR transmittance spectra of a pharmaceutical tablet were described in Dyrby et al. (2002) and publicly available at http://www.models.life.ku.dk/Tablets. This tablet dataset consists of 310 samples measured in the range of 7,000–10,500 cm^−1^ with a resolution of 16 cm^−1^ i.e., a total number of 404 variables per sample. The objective of the analysis was to predict the API content of the tablet. The content of API in the tablets (% w/w) was assayed by high performance liquid chromatography (HPLC). The tablet dataset was supplied in Data Sheet 1. This dataset was divided into two groups: 207 and 103 samples for training and validation with Kennard-Stone (KS) algorithm, respectively.

#### Lonicera japonica

The NIR spectral dataset of *Lonicera japonica* has been reported previously (Wu et al., 2012). The data consisted of 216 samples with 2,800 variables in the range of 1,100–2,500 nm that measured on an XDS rapid liquid analyzer with VISION software in the transmission mode (Foss NIR Systems, Silver Spring, MD, USA). NIR spectra of *Lonicera japonica* solution obtained from ethanol precipitation process, were measured to estimate chlorogenic acid content. HPLC was used as the reference method for chlorogenic acid determination as recommended by the Chinese Pharmacopoeia (CHP, 2010 Edition) for *Lonicera japonica* monograph. The dataset of *Lonicera japonica* was supplied in Data Sheet 2. In this study, the training data consisted of 144 samples and the remaining 72 samples were used for validation.

#### Multivariate data analyses

The spectral pretreatment of data was performed using chemometric tool in this study (SIMCA P + 11.5, Umetrics, Sweden). Data analysis was conducted using Unscrambler 9.7 software package (Camo Software AS, Norway) and Matlab version 7.0 (MathWorks Inc., USA). Some of the algorithms were developed by Norgaard et al., readily downloadable from http://www.models.life.ku.dk/iToolbox.

#### Multivariate calibration

A procedure for the development and optimization of multivariate calibration models using processing trajectory is summarized in Figure 2. The rationale behind this approach is that there was more than one path to obtain good model with different parameter combinations. Thus, the procedure was used to track and evaluate modeling processes with different parameters including spectral pretreatments, variable selections, latent factors, and calibration methods. The evaluation indexes of model includes RMSEP and RPD.

## Result and discussion

### Raw spectra

**Figure 1:**
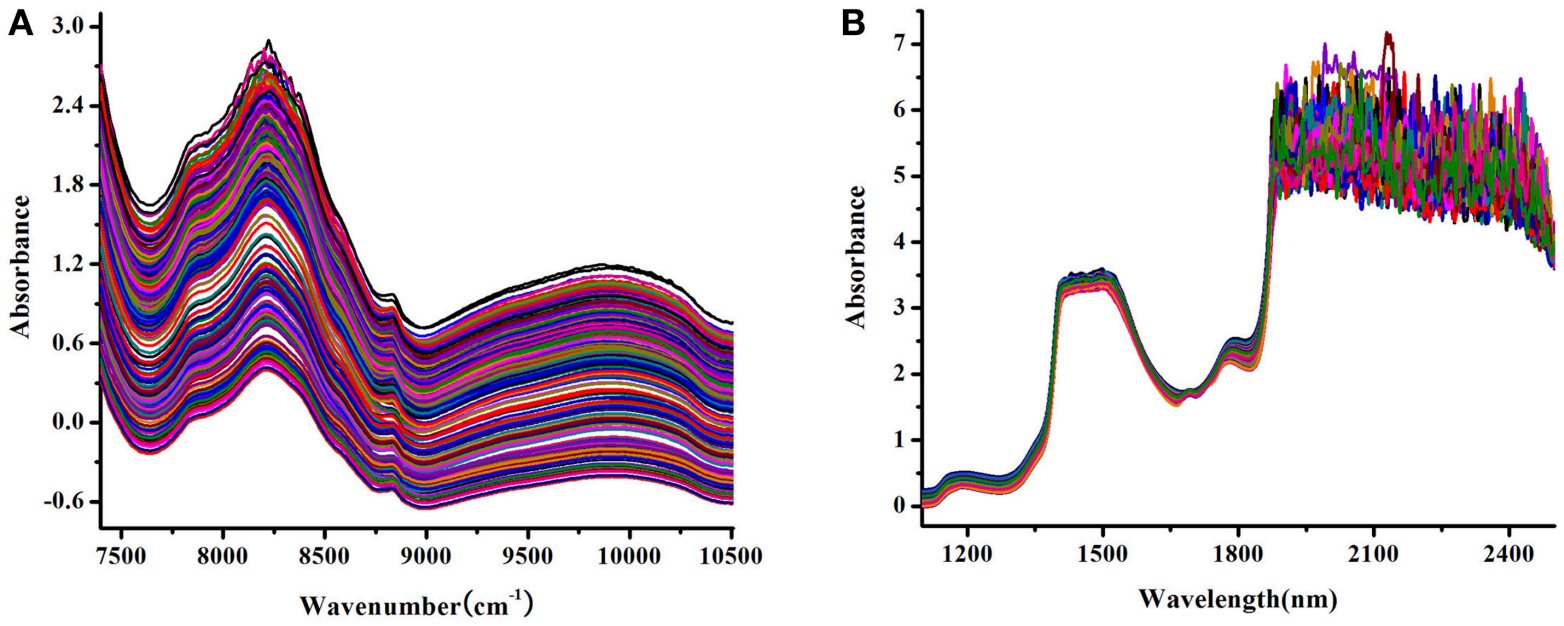
Raw NIR spectra of tablet samples **(A)** and *Lonicera japonica* solution in ethanol precipitation process **(B)**.

The raw NIR spectra of the tablet and *Lonicera japonica* solution were shown in Figure 1, which represent their characteristic peak locations regarding the active substance in each spectral dataset. In the NIR transmittance spectra of tablet (Figure 1A), there were several broad peaks located at around 10,000, 8,830, 8,200, and 7,840 cm^−1^, which originated from several components in the corresponding drug tablet. In addition, there were large fluctuations in the combined region of fundamental vibrations in the raw spectra of *Lonicera japonica* solution. Therefore, the spectral region of 1,100–1,900 nm was selected.

### Processing trajectory of PLS model

**Figure 2:**
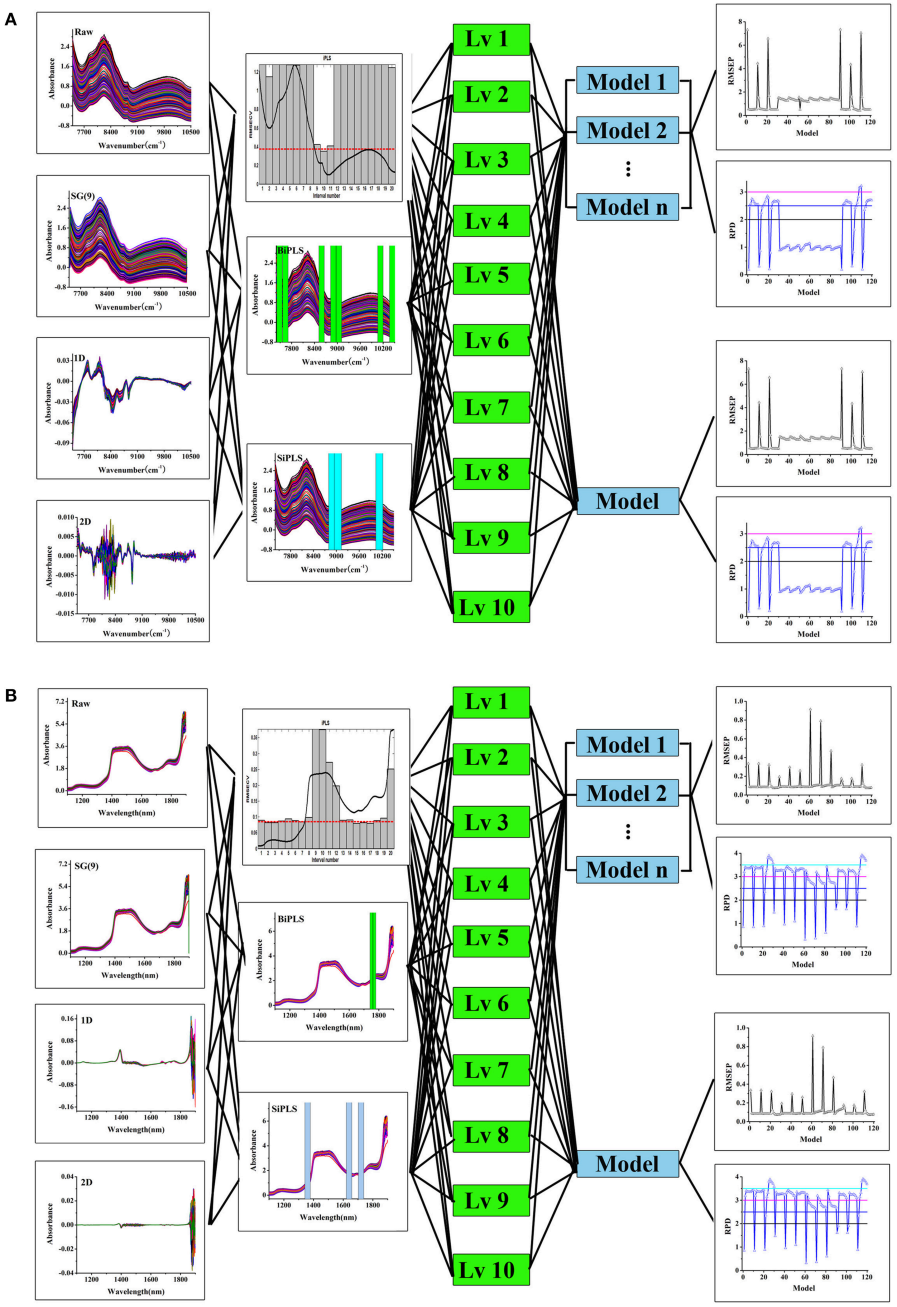
The processing trajectory and assessment of PLS and Bagging-PLS model, tablet samples **(A)** and *Lonicera japonica* solution in ethanol precipitation process **(B)**.

The modeling procedure using processing trajectory was showed in Figure 2. Taking the tablet dataset as an example, the data set were split in to calibration and validation sets and the spectra were preprocessed using different methods including first derivative (1st), second derivative (2nd) and Savitzky-Golay smoothing with 9 points [SG(9)]. The iPLS, BiPLS and SiPLS were then used to select variables. Finally, the PLS and Bagging-PLS models were developed with latent factors from 1 to 10. Both RPD and RMSEP were calculated to evaluate the model. Figure 2 showed different modeling paths and model results. The parameters for PLS and Bagging-PLS models of API in tablet and chlorogenic acid of *Lonicera japonica* solution were shown in Tables S1, S2.

The RPD and RMSEP had similar trends in PLS and Bagging-PLS models. In Figure 2A, the RMSEP decreased with increasing latent factor coupled with different pretreatment methods and variables selections. The RPD also increased with an increase of small latent factors. However, when the latent variable was greater than a certain value, the RPD became smaller. Variances in RMSEP and RPD indexes were not obvious when using 1st and 2nd derivative preprocessed spectra. Other pretreatment methods were superior to 1st and 2nd derivative processing. The model for *Lonicera japonica* dataset is shown in Figure 2B. Similar results were found for the tablet dataset. The model results of other pretreatment methods were also better than 2nd derivative processing.

**Figure 3:**
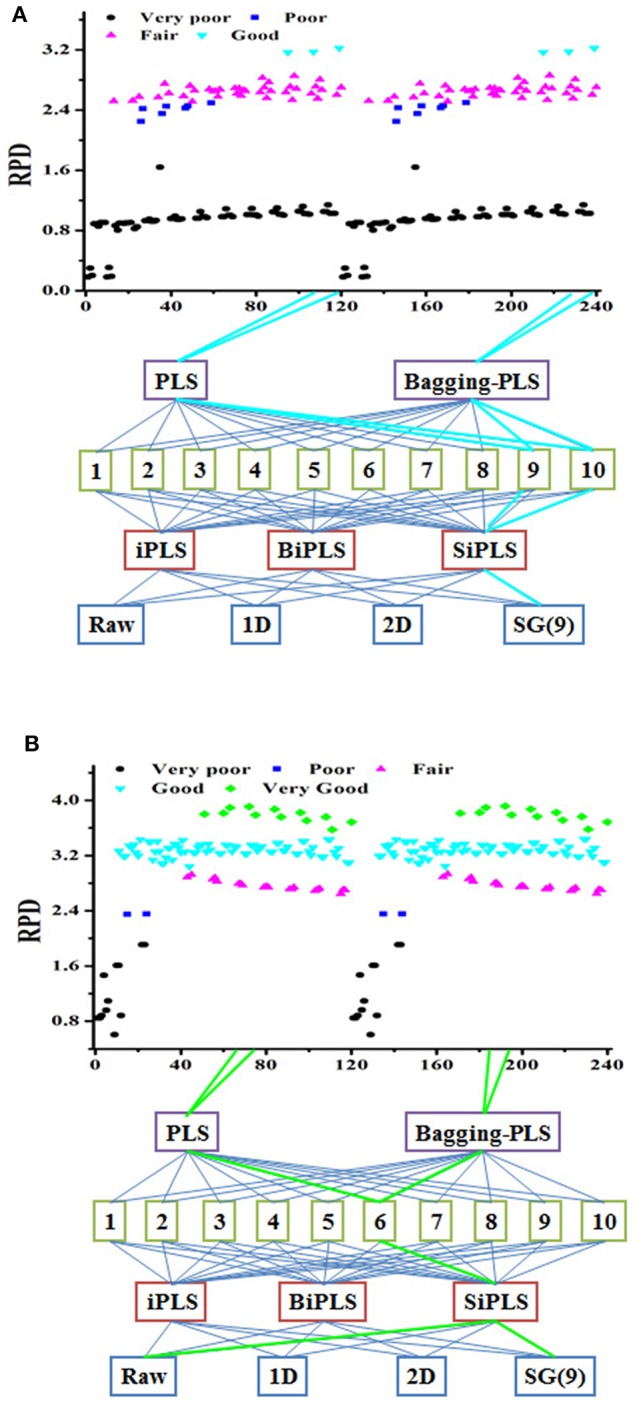
The processing trajectory of PLS and Bagging-PLS model tablet samples **(A)** and *Lonicera japonica* solution in ethanol precipitation process **(B)**.

Moreover, this finding indicates that more than one modeling path could ensure a successful model. Data obtained from different modeling paths and model classification were shown in Figure 3. There were six good models with RPD between 3 and 3.5 (Figure 3A), and some very good model paths with RPD values greater than 3.5 (Figure 3B). In the previous modeling process routine, the parameters were optimized one at a time according to the resultant prediction accuracy. This was a poor approach to path modeling vs. step-by-step parameter optimization (Table S3). The optimal parameters of the API model obtained step-by-step optimized were the raw spectra and iPLS-selecting variable under 3 latent factors. The model performance was fair. However, the result of processing trajectory showed that six good models could be obtained by combination of SG(9) pretreatment and BiPLS-selecting variables.

### Development and validation of calibration models

The best nonsystematic parameter combination for the chlorogenic acid Bagging-PLS model was raw spectra and iPLS or BiPLS variables selection under 2 latent factors. The model performance was good. However, there were 24 very good models with different systematic parameter combinations in the result of processing trajectory. The best parameter combination of the chlorogenic acid model was that the model was developed by Bagging-PLS with SG(9) spectral pretreatment and SiPLS-selecting variables under 6 factors. It demonstrated that the model obtained through the processing trajectory was better than that step-by-step optimized. It means that the optimal systematic model parameter combination can be obtained via the processing trajectory and bagging ensemble modeling techniques, and variable assignment could improve prediction accuracy and robustness.

**Figure 4:**
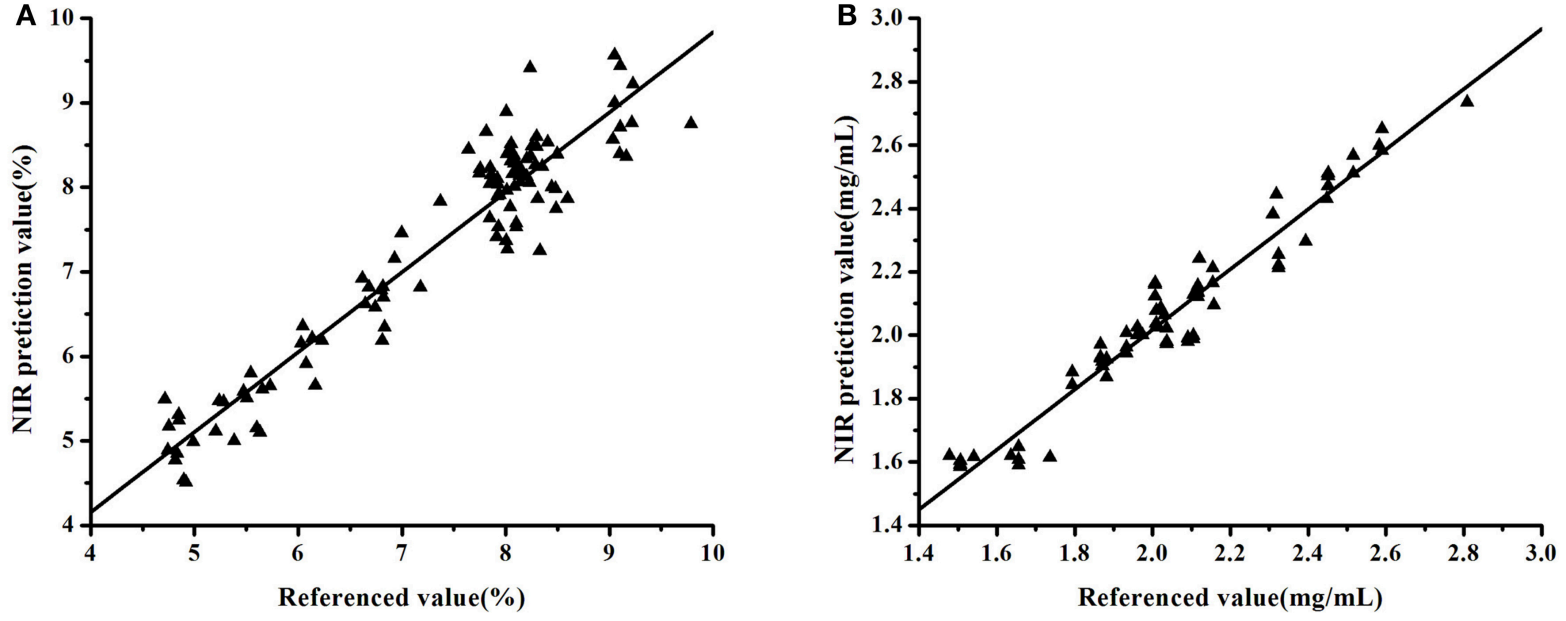
Correlation between the prediction and reference values of the datasets: tablet samples **(A)** and *Lonicera japonica* solution in ethanol precipitation process **(B)**.

The model validity was evaluated in terms of RMSEP and RPD values. Taking the tablet dataset as an example, Figure 2A showed that the model established using Bagging-PLS with SG(9) pretreatment and BiPLS-selecting variables under 10 latent factors had the best performance. The RMSEP and RPD values of the validation set were 0.4126% and 3.2234, respectively. In contrast, the RMSEP and RPD of the model step-by-step optimized were 0.5164% and 2.5755, respectively. These results also showed that the model developed with Bagging-PLS had a good predictive performance. Similarly, the model of *Lonicera japonica* solution was developed using Bagging-PLS with SG(9) spectral pretreatment and SiPLS-selecting variables under 6 latent factors. The RMSEP and RPD were 0.0728 mg/mL and 3.9166, respectively. The RMSEP and RPD of the model step-by-step optimized were 0.0891% and 3.1966, respectively. Figure 4 presents the data obtained with Bagging-PLS models using the two datasets. The prediction values reasonably agreed with HPLC results. The parameters indicated that NIRS could be used for the determination of API in tablets and chlorogenic acid of *Lonicera japonica* solution in ethanol precipitation process.

## Conclusion

We proposed processing trajectory to optimize the parameters of multivariate calibration such as spectral pretreatment, latent factors, variable selection and calibration methods. The models were developed using PLS and Bagging-PLS with different spectral pretreatments and variable selection methods under different latent factors. The chemometric indicators (RMSEP and RPD) were used to evaluated the model. The different PLS and Bagging-PLS models were used to quantify the API in tablets and chlorogenic acid of *Lonicera japonica* solution in ethanol precipitation process. The result illustrated that the processing trajectory has great advantages and feasibility in the development and optimization of multivariate calibration models and the effectiveness of bagging model and variable selection to improve prediction accuracy and robustness.

In conclusion, the application of processing trajectory for model optimization shows excellent results to develop a reliable and robust model. The proposed should be translated into an algorithm to be integrated into PLS software, helping to obtain better models.

## Author contributions

YQ and ZW conceived and designed the study. NZ performed the experiment with the help of LM, XH, and XL. NZ and ZW wrote the manuscript. All authors read and approved the final manuscript.

### Conflict of interest statement

The authors declare that the research was conducted in the absence of any commercial or financial relationships that could be construed as a potential conflict of interest.
