# Supplementary material for: Pharmaceutical Analysis Model Robustness From Bagging-PLS and PLS Using Systematic Tracking Mapping
Source: Front Chem. 2018 Jul 6;6:262. doi: 10.3389/fchem.2018.00262 (PMC6043861; doi:10.3389/fchem.2018.00262)
Supplement: Supplementary file 1 [file Table_1.DOC]

**Supplementary Table S1.** The parameters of PLS and Bagging-PLS models of tablet via different spectra pretreatment and variables selections.

| Model | Pretreamt | Variable selection | Latent factor | PLS | | | Bagging-PLS | | |
| --- | --- | --- | --- | --- | --- | --- | --- | --- | --- |
|  |  |  |  | RMSEP | RPD | Classification | RMSEP | RPD | Classification |
| 1 | Raw | iPLS | 1 | 7.3183 | 0.1817 | Very Poor | 7.3162 | 0.1818 | Very Poor |
| 2 |  |  | 2 | 0.5281 | 2.5185 | Fair | 0.5280 | 2.5190 | Fair |
| 3 |  |  | 3 | 0.5165 | 2.5742 | Fair | 0.5164 | 2.5755 | Fair |
| 4 |  |  | 4 | 0.4838 | 2.7480 | Fair | 0.4837 | 2.7495 | Fair |
| 5 |  |  | 5 | 0.4875 | 2.7205 | Fair | 0.4887 | 2.7216 | Fair |
| 6 |  |  | 6 | 0.5030 | 2.6498 | Fair | 0.5016 | 2.6512 | Fair |
| 7 |  |  | 7 | 0.5198 | 2.5891 | Fair | 0.5133 | 2.5908 | Fair |
| 8 |  |  | 8 | 0.5207 | 2.5564 | Fair | 0.5200 | 2.5577 | Fair |
| 9 |  |  | 9 | 0.5253 | 2.5315 | Fair | 0.5252 | 2.5323 | Fair |
| 10 |  |  | 10 | 0.5190 | 2.5494 | Fair | 0.5210 | 2.5526 | Fair |
| 11 | Raw | BiPLS | 1 | 4.4452 | 0.2992 | Very Poor | 4.4435 | 0.2993 | Very Poor |
| 12 |  |  | 2 | 1.5364 | 0.8657 | Very Poor | 1.5360 | 0.8659 | Very Poor |
| 13 |  |  | 3 | 0.5931 | 2.2459 | Poor | 0.5917 | 2.2479 | Poor |
| 14 |  |  | 4 | 0.5429 | 2.4489 | Poor | 0.5426 | 2.4511 | Poor |
| 15 |  |  | 5 | 0.5301 | 2.5115 | Fair | 0.5291 | 2.5136 | Fair |
| 16 |  |  | 6 | 0.5199 | 2.5770 | Fair | 0.5160 | 2.5777 | Fair |
| 17 |  |  | 7 | 0.5020 | 2.6610 | Fair | 0.4994 | 2.6630 | Fair |
| 18 |  |  | 8 | 0.4781 | 2.7687 | Fair | 0.4801 | 2.7702 | Fair |
| 19 |  |  | 9 | 0.4638 | 2.8511 | Fair | 0.4656 | 2.8565 | Fair |
| 20 |  |  | 10 | 0.4790 | 2.8042 | Fair | 0.4739 | 2.8063 | Fair |
| 21 | Raw | SiPLS | 1 | 6.5753 | 0.2023 | Very Poor | 6.5707 | 0.2024 | Very Poor |
| 22 |  |  | 2 | 1.6585 | 0.8020 | Very Poor | 1.6581 | 0.8021 | Very Poor |
| 23 |  |  | 3 | 0.5507 | 2.4162 | Poor | 0.5504 | 2.4265 | Poor |
| 24 |  |  | 4 | 0.5074 | 2.6206 | Fair | 0.5123 | 2.6210 | Fair |
| 25 |  |  | 5 | 0.5018 | 2.6582 | Fair | 0.4998 | 2.6610 | Fair |
| 26 |  |  | 6 | 0.5012 | 2.6713 | Fair | 0.4969 | 2.6765 | Fair |
| 27 |  |  | 7 | 0.5060 | 2.6512 | Fair | 0.5005 | 2.6573 | Fair |
| 28 |  |  | 8 | 0.5005 | 2.6501 | Fair | 0.5008 | 2.6559 | Fair |
| 29 |  |  | 9 | 0.4953 | 2.6810 | Fair | 0.4954 | 2.6849 | Fair |
| 30 |  |  | 10 | 0.5051 | 2.6653 | Fair | 0.4989 | 2.6661 | Fair |
| 31 | 1st | iPLS | 1 | 1.5012 | 0.8863 | Very Poor | 1.4998 | 0.8868 | Very Poor |
| 32 |  |  | 2 | 1.4925 | 0.8958 | Very Poor | 1.4828 | 0.8969 | Very Poor |
| 33 |  |  | 3 | 1.4339 | 0.9283 | Very Poor | 1.4322 | 0.9286 | Very Poor |
| 34 |  |  | 4 | 1.3817 | 0.9562 | Very Poor | 1.3904 | 0.9566 | Very Poor |
| 35 |  |  | 5 | 1.3887 | 0.9614 | Very Poor | 1.3830 | 0.9617 | Very Poor |
| 36 |  |  | 6 | 1.3419 | 0.9794 | Very Poor | 1.3564 | 0.9805 | Very Poor |
| 37 |  |  | 7 | 1.2761 | 1.0075 | Very Poor | 1.3188 | 1.0084 | Very Poor |
| 38 |  |  | 8 | 1.2800 | 1.0454 | Very Poor | 1.2714 | 1.0461 | Very Poor |
| 39 |  |  | 9 | 1.2685 | 1.0531 | Very Poor | 1.2624 | 1.0535 | Very Poor |
| 40 |  |  | 10 | 1.2758 | 1.0508 | Very Poor | 1.2651 | 1.0513 | Very Poor |
| 41 | 1st | Bipls | 1 | 1.5012 | 0.8863 | Very Poor | 1.4998 | 0.8868 | Very Poor |
| 42 |  |  | 2 | 1.4925 | 0.8958 | Very Poor | 1.4828 | 0.8969 | Very Poor |
| 43 |  |  | 3 | 1.4339 | 0.9283 | Very Poor | 1.4322 | 0.9286 | Very Poor |
| 44 |  |  | 4 | 1.3817 | 0.9562 | Very Poor | 1.3904 | 0.9566 | Very Poor |
| 45 |  |  | 5 | 1.3887 | 0.9614 | Very Poor | 1.3830 | 0.9617 | Very Poor |
| 46 |  |  | 6 | 1.3419 | 0.9794 | Very Poor | 1.3564 | 0.9805 | Very Poor |
| 47 |  |  | 7 | 1.2761 | 1.0075 | Very Poor | 1.3188 | 1.0084 | Very Poor |
| 48 |  |  | 8 | 1.2800 | 1.0454 | Very Poor | 1.2714 | 1.0461 | Very Poor |
| 49 |  |  | 9 | 1.2685 | 1.0531 | Very Poor | 1.2624 | 1.0535 | Very Poor |
| 50 |  |  | 10 | 1.2758 | 1.0508 | Very Poor | 1.2651 | 1.0513 | Very Poor |
| 51 | 1st | SiPLS | 1 | 1.5580 | 0.8541 | Very Poor | 1.5568 | 0.8543 | Very Poor |
| 52 |  |  | 2 | 1.4917 | 0.8916 | Very Poor | 0.4915 | 0.8917 | Very Poor |
| 53 |  |  | 3 | 1.4071 | 0.9468 | Very Poor | 1.4037 | 0.9475 | Very Poor |
| 54 |  |  | 4 | 1.3466 | 0.9893 | Very Poor | 1.3441 | 0.9895 | Very Poor |
| 55 |  |  | 5 | 1.2554 | 1.0499 | Very Poor | 1.2654 | 1.0511 | Very Poor |
| 56 |  |  | 6 | 1.2204 | 1.0874 | Very Poor | 1.2223 | 1.0881 | Very Poor |
| 57 |  |  | 7 | 1.2332 | 1.0903 | Very Poor | 1.2191 | 1.0910 | Very Poor |
| 58 |  |  | 8 | 1.2192 | 1.0982 | Very Poor | 1.2098 | 1.0994 | Very Poor |
| 59 |  |  | 9 | 1.1953 | 1.1188 | Very Poor | 1.1885 | 1.1190 | Very Poor |
| 60 |  |  | 10 | 1.1707 | 1.1385 | Very Poor | 1.1678 | 1.1389 | Very Poor |
| 61 | 2nd | iPLS | 1 | 1.4736 | 0.9027 | Very Poor | 1.4729 | 0.9027 | Very Poor |
| 62 |  |  | 2 | 1.4756 | 0.9018 | Very Poor | 1.4745 | 0.9020 | Very Poor |
| 63 |  |  | 3 | 1.4452 | 0.9214 | Very Poor | 1.4429 | 0.9217 | Very Poor |
| 64 |  |  | 4 | 1.4206 | 0.9433 | Very Poor | 1.4093 | 0.9437 | Very Poor |
| 65 |  |  | 5 | 1.3661 | 0.9811 | Very Poor | 1.3547 | 0.9818 | Very Poor |
| 66 |  |  | 6 | 1.3396 | 1.0004 | Very Poor | 1.3280 | 1.0015 | Very Poor |
| 67 |  |  | 7 | 1.3257 | 1.0072 | Very Poor | 1.3199 | 1.0076 | Very Poor |
| 68 |  |  | 8 | 1.3246 | 1.0133 | Very Poor | 1.3106 | 1.0148 | Very Poor |
| 69 |  |  | 9 | 1.3145 | 1.0198 | Very Poor | 1.3031 | 1.0206 | Very Poor |
| 70 |  |  | 10 | 1.3100 | 1.0245 | Very Poor | 1.2973 | 1.0252 | Very Poor |
| 71 | 2nd | BiPLS | 1 | 1.4736 | 0.9027 | Very Poor | 1.4729 | 0.9027 | Very Poor |
| 72 |  |  | 2 | 1.4756 | 0.9018 | Very Poor | 1.4745 | 0.9020 | Very Poor |
| 73 |  |  | 3 | 1.4452 | 0.9214 | Very Poor | 1.4429 | 0.9217 | Very Poor |
| 74 |  |  | 4 | 1.4206 | 0.9433 | Very Poor | 1.4093 | 0.9437 | Very Poor |
| 75 |  |  | 5 | 1.3661 | 0.9811 | Very Poor | 1.3547 | 0.9818 | Very Poor |
| 76 |  |  | 6 | 1.3396 | 1.0004 | Very Poor | 1.3280 | 1.0015 | Very Poor |
| 77 |  |  | 7 | 1.3257 | 1.0072 | Very Poor | 1.3199 | 1.0076 | Very Poor |
| 78 |  |  | 8 | 1.3246 | 1.0133 | Very Poor | 1.3106 | 1.0148 | Very Poor |
| 79 |  |  | 9 | 1.3145 | 1.0198 | Very Poor | 1.3031 | 1.0206 | Very Poor |
| 80 |  |  | 10 | 1.3100 | 1.0245 | Very Poor | 1.2973 | 1.0252 | Very Poor |
| 81 | 2nd | SiPLS | 1 | 1.4734 | 0.9028 | Very Poor | 1.4727 | 0.9031 | Very Poor |
| 82 |  |  | 2 | 1.4760 | 0.9062 | Very Poor | 1.4758 | 0.9073 | Very Poor |
| 83 |  |  | 3 | 1.4208 | 0.9313 | Very Poor | 1.4272 | 0.9319 | Very Poor |
| 84 |  |  | 4 | 1.4030 | 0.9537 | Very Poor | 1.3938 | 0.9542 | Very Poor |
| 85 |  |  | 5 | 1.3708 | 0.9695 | Very Poor | 1.3707 | 0.9703 | Very Poor |
| 86 |  |  | 6 | 1.3677 | 0.9802 | Very Poor | 1.3560 | 0.9808 | Very Poor |
| 87 |  |  | 7 | 1.3567 | 0.9911 | Very Poor | 1.3411 | 0.9917 | Very Poor |
| 88 |  |  | 8 | 1.3358 | 1.0022 | Very Poor | 1.3258 | 1.0032 | Very Poor |
| 89 |  |  | 9 | 1.3231 | 1.0130 | Very Poor | 1.3118 | 1.0139 | Very Poor |
| 90 |  |  | 10 | 1.2956 | 1.0271 | Very Poor | 1.2945 | 1.0274 | Very Poor |
| 91 | SG(9) | iPLS | 1 | 7.3431 | 0.1811 | Very Poor | 7.3406 | 0.1812 | Very Poor |
| 92 |  |  | 2 | 0.5277 | 2.5209 | Fair | 0.5276 | 2.5210 | Fair |
| 93 |  |  | 3 | 0.5178 | 2.5687 | Fair | 0.5177 | 2.5690 | Fair |
| 94 |  |  | 4 | 0.5164 | 2.5816 | Fair | 0.5151 | 2.5820 | Fair |
| 95 |  |  | 5 | 0.4972 | 2.6753 | Fair | 0.4970 | 2.6758 | Fair |
| 96 |  |  | 6 | 0.4930 | 2.6903 | Fair | 0.4938 | 2.6934 | Fair |
| 97 |  |  | 7 | 0.5056 | 2.6354 | Fair | 0.5046 | 2.6359 | Fair |
| 98 |  |  | 8 | 0.5053 | 2.6346 | Fair | 0.5047 | 2.6350 | Fair |
| 99 |  |  | 9 | 0.5073 | 2.6175 | Fair | 0.5074 | 2.6212 | Fair |
| 100 |  |  | 10 | 0.5100 | 2.6012 | Fair | 0.5110 | 2.6029 | Fair |
| 101 | SG(9) | BiPLS | 1 | 4.3605 | 0.3051 | Very Poor | 4.3583 | 0.3052 | Very Poor |
| 102 |  |  | 2 | 1.6132 | 0.8244 | Very Poor | 1.6130 | 0.8245 | Very Poor |
| 103 |  |  | 3 | 0.8159 | 1.6381 | Very Poor | 0.8112 | 1.6395 | Very Poor |
| 104 |  |  | 4 | 0.5485 | 2.4254 | Poor | 0.5481 | 2.4266 | Poor |
| 105 |  |  | 5 | 0.5339 | 2.4946 | Poor | 0.5331 | 2.4948 | Poor |
| 106 |  |  | 6 | 0.5098 | 2.6168 | Fair | 0.5081 | 2.6174 | Fair |
| 107 |  |  | 7 | 0.4716 | 2.8274 | Fair | 0.4703 | 2.8280 | Fair |
| 108 |  |  | 8 | 0.4212 | 3.1661 | Good | 0.4199 | 3.1677 | Good |
| 109 |  |  | 9 | 0.4265 | 3.1727 | Good | 0.4190 | 3.1742 | Good |
| 110 |  |  | 10 | 0.4162 | 3.2230 | Good | 0.4126 | 3.2234 | Good |
| 111 | SG(9) | SiPLS | 1 | 7.0596 | 0.1884 | Very Poor | 7.0570 | 0.1885 | Very Poor |
| 112 |  |  | 2 | 1.5678 | 0.8482 | Very Poor | 1.5677 | 0.8484 | Very Poor |
| 113 |  |  | 3 | 0.5659 | 2.3503 | Poor | 0.5657 | 2.3508 | Poor |
| 114 |  |  | 4 | 0.5498 | 2.4540 | Poor | 0.5417 | 2.4551 | Poor |
| 115 |  |  | 5 | 0.5027 | 2.6475 | Fair | 0.5018 | 2.6502 | Fair |
| 116 |  |  | 6 | 0.4974 | 2.6873 | Fair | 0.4947 | 2.6886 | Fair |
| 117 |  |  | 7 | 0.4960 | 2.6974 | Fair | 0.4928 | 2.6988 | Fair |
| 118 |  |  | 8 | 0.4931 | 2.7077 | Fair | 0.4910 | 2.7086 | Fair |
| 119 |  |  | 9 | 0.4963 | 2.7096 | Fair | 0.4904 | 2.7121 | Fair |
| 120 |  |  | 10 | 0.4912 | 2.7000 | Fair | 0.4923 | 2.7013 | Fair |

***The unit of RMSEP is mg/mg (%).**
