# Supplementary material for: Pharmaceutical Analysis Model Robustness From Bagging-PLS and PLS Using Systematic Tracking Mapping
Source: Front Chem. 2018 Jul 6;6:262. doi: 10.3389/fchem.2018.00262 (PMC6043861; doi:10.3389/fchem.2018.00262)
Supplement: Supplementary file 2 [file Table_2.DOC]

**Supplementary Table S2. The parameters of PLS and Bagging-PLS models of *Lonicera japonica*** via different spectra pretreatment and variables selections.

| Model | Pretreamt | Variable selection | latent factor | PLS | | | Bagging-PLS | | |
| --- | --- | --- | --- | --- | --- | --- | --- | --- | --- |
|  |  |  |  | RMSEP | RPD | Classification | RMSEP | RPD | Classification |
| 1 | Raw | iPLS | 1 | 0.3360 | 0.8478 | Very poor | 0.3359 | 0.8483 | Very poor |
| 2 |  |  | 2 | 0.0893 | 3.1917 | Good | 0.0891 | 3.1966 | Good |
| 3 |  |  | 3 | 0.0837 | 3.4026 | Good | 0.0837 | 3.4036 | Good |
| 4 |  |  | 4 | 0.0849 | 3.3659 | Good | 0.0846 | 3.3674 | Good |
| 5 |  |  | 5 | 0.0849 | 3.3576 | Good | 0.0848 | 3.3586 | Good |
| 6 |  |  | 6 | 0.0850 | 3.3557 | Good | 0.0849 | 3.3563 | Good |
| 7 |  |  | 7 | 0.0847 | 3.3537 | Good | 0.0849 | 3.3546 | Good |
| 8 |  |  | 8 | 0.0843 | 3.3832 | Good | 0.0842 | 3.3839 | Good |
| 9 |  |  | 9 | 0.0840 | 3.4102 | Good | 0.0835 | 3.4112 | Good |
| 10 |  |  | 10 | 0.0833 | 3.4366 | Good | 0.0828 | 3.4411 | Good |
| 11 | Raw | BiPLS | 1 | 0.3360 | 0.8478 | Very poor | 0.3359 | 0.8483 | Very poor |
| 12 |  |  | 2 | 0.0893 | 3.1917 | Good | 0.0891 | 3.1966 | Good |
| 13 |  |  | 3 | 0.0837 | 3.4026 | Good | 0.0837 | 3.4036 | Good |
| 14 |  |  | 4 | 0.0849 | 3.3659 | Good | 0.0846 | 3.3674 | Good |
| 15 |  |  | 5 | 0.0849 | 3.3576 | Good | 0.0848 | 3.3586 | Good |
| 16 |  |  | 6 | 0.0850 | 3.3557 | Good | 0.0849 | 3.3563 | Good |
| 17 |  |  | 7 | 0.0847 | 3.3537 | Good | 0.0849 | 3.3546 | Good |
| 18 |  |  | 8 | 0.0843 | 3.3832 | Good | 0.0842 | 3.3839 | Good |
| 19 |  |  | 9 | 0.0840 | 3.4102 | Good | 0.0835 | 3.4112 | Good |
| 20 |  |  | 10 | 0.0833 | 3.4366 | Good | 0.0828 | 3.4411 | Good |
| 21 | Raw | SiPLS | 1 | 0.3233 | 0.8827 | Very poor | 0.3222 | 0.8845 | Very poor |
| 22 |  |  | 2 | 0.1214 | 2.3479 | Poor | 0.1213 | 2.3495 | Poor |
| 23 |  |  | 3 | 0.0910 | 3.1358 | Good | 0.0909 | 3.1361 | Good |
| 24 |  |  | 4 | 0.0859 | 3.3183 | Good | 0.0858 | 3.3196 | Good |
| 25 |  |  | 5 | 0.0748 | 3.7987 | Very good | 0.0747 | 3.8122 | Very good |
| 26 |  |  | 6 | 0.0729 | 3.8932 | Very good | 0.0731 | 3.8962 | Very good |
| 27 |  |  | 7 | 0.0754 | 3.7874 | Very good | 0.0752 | 3.7885 | Very good |
| 28 |  |  | 8 | 0.0750 | 3.7599 | Very good | 0.0757 | 3.7661 | Very good |
| 29 |  |  | 9 | 0.0761 | 3.7041 | Very good | 0.0768 | 3.7111 | Very good |
| 30 |  |  | 10 | 0.0810 | 3.5751 | Very good | 0.0796 | 3.5790 | Very good |
| 31 | 1st | iPLS | 1 | 0.1945 | 1.4665 | Very poor | 0.1942 | 1.4670 | Very poor |
| 32 |  |  | 2 | 0.0853 | 3.3406 | Good | 0.0852 | 3.3427 | Good |
| 33 |  |  | 3 | 0.0879 | 3.2548 | Good | 0.0875 | 3.2562 | Good |
| 34 |  |  | 4 | 0.0877 | 3.2430 | Good | 0.0877 | 3.2472 | Good |
| 35 |  |  | 5 | 0.0878 | 3.2540 | Good | 0.0875 | 3.2570 | Good |
| 36 |  |  | 6 | 0.0873 | 3.2568 | Good | 0.0874 | 3.2600 | Good |
| 37 |  |  | 7 | 0.0872 | 3.2682 | Good | 0.0871 | 3.2724 | Good |
| 38 |  |  | 8 | 0.0870 | 3.2709 | Good | 0.0870 | 3.2744 | Good |
| 39 |  |  | 9 | 0.0874 | 3.2587 | Good | 0.0874 | 3.2602 | Good |
| 40 |  |  | 10 | 0.0882 | 3.2513 | Good | 0.0875 | 3.2564 | Good |
| 41 | 1st | BiPLS | 1 | 0.2969 | 0.9569 | Very poor | 0.2965 | 0.9611 | Very poor |
| 42 |  |  | 2 | 0.0860 | 3.3151 | Good | 0.0859 | 3.3160 | Good |
| 43 |  |  | 3 | 0.0839 | 3.4036 | Good | 0.0837 | 3.4045 | Good |
| 44 |  |  | 4 | 0.0833 | 3.4221 | Good | 0.0832 | 3.4234 | Good |
| 45 |  |  | 5 | 0.0850 | 3.3673 | Good | 0.0846 | 3.3697 | Good |
| 46 |  |  | 6 | 0.0856 | 3.3357 | Good | 0.0854 | 3.3384 | Good |
| 47 |  |  | 7 | 0.0866 | 3.3111 | Good | 0.0860 | 3.3115 | Good |
| 48 |  |  | 8 | 0.0869 | 3.2783 | Good | 0.0868 | 3.2810 | Good |
| 49 |  |  | 9 | 0.0890 | 3.2062 | Good | 0.0888 | 3.2090 | Good |
| 50 |  |  | 10 | 0.0900 | 3.1880 | Good | 0.0894 | 3.1887 | Good |
| 51 | 1st | SiPLS | 1 | 0.2607 | 1.0926 | Very poor | 0.2606 | 1.0934 | Very poor |
| 52 |  |  | 2 | 0.0848 | 3.3576 | Good | 0.0849 | 3.3590 | Good |
| 53 |  |  | 3 | 0.0871 | 3.2598 | Good | 0.0874 | 3.2609 | Good |
| 54 |  |  | 4 | 0.0877 | 3.2554 | Good | 0.0875 | 3.2566 | Good |
| 55 |  |  | 5 | 0.0873 | 3.2835 | Good | 0.0867 | 3.2847 | Good |
| 56 |  |  | 6 | 0.0855 | 3.3239 | Good | 0.0856 | 3.3269 | Good |
| 57 |  |  | 7 | 0.0859 | 3.3310 | Good | 0.0854 | 3.3348 | Good |
| 58 |  |  | 8 | 0.0856 | 3.3285 | Good | 0.0855 | 3.3323 | Good |
| 59 |  |  | 9 | 0.0860 | 3.3187 | Good | 0.0857 | 3.3236 | Good |
| 60 |  |  | 10 | 0.0868 | 3.3067 | Good | 0.0861 | 3.3082 | Good |
| 61 | 2nd | iPLS | 1 | 0.9160 | 0.3109 | Very poor | 0.9144 | 0.3116 | Very poor |
| 62 |  |  | 2 | 0.0879 | 3.2425 | Good | 0.0878 | 3.2436 | Good |
| 63 |  |  | 3 | 0.0943 | 3.0897 | Good | 0.0921 | 3.0942 | Good |
| 64 |  |  | 4 | 0.1014 | 2.8850 | Fair | 0.0986 | 2.8886 | Fair |
| 65 |  |  | 5 | 0.1014 | 2.8547 | Fair | 0.0998 | 2.8564 | Fair |
| 66 |  |  | 6 | 0.1044 | 2.7882 | Fair | 0.1020 | 2.7950 | Fair |
| 67 |  |  | 7 | 0.1101 | 2.7384 | Fair | 0.1039 | 2.7419 | Fair |
| 68 |  |  | 8 | 0.1119 | 2.7140 | Fair | 0.1050 | 2.7147 | Fair |
| 69 |  |  | 9 | 0.1134 | 2.6889 | Fair | 0.1060 | 2.6893 | Fair |
| 70 |  |  | 10 | 0.1177 | 2.6448 | Fair | 0.1077 | 2.6463 | Fair |
| 71 | 2nd | BiPLS | 1 | 0.7919 | 0.3594 | Very poor | 0.7908 | 0.3603 | Very poor |
| 72 |  |  | 2 | 0.0891 | 3.1963 | Good | 0.0891 | 3.1972 | Good |
| 73 |  |  | 3 | 0.0936 | 3.0865 | Good | 0.0922 | 3.0896 | Good |
| 74 |  |  | 4 | 0.0943 | 3.0409 | Good | 0.0936 | 3.0452 | Good |
| 75 |  |  | 5 | 0.1029 | 2.8787 | Fair | 0.0989 | 2.8814 | Fair |
| 76 |  |  | 6 | 0.1056 | 2.7940 | Fair | 0.1017 | 2.8023 | Fair |
| 77 |  |  | 7 | 0.1053 | 2.7534 | Fair | 0.1032 | 2.7609 | Fair |
| 78 |  |  | 8 | 0.1097 | 2.7096 | Fair | 0.1051 | 2.7114 | Fair |
| 79 |  |  | 9 | 0.1086 | 2.7050 | Fair | 0.1051 | 2.7118 | Fair |
| 80 |  |  | 10 | 0.1081 | 2.7036 | Fair | 0.1053 | 2.7056 | Fair |
| 81 | 2nd | SiPLS | 1 | 0.4725 | 0.6035 | Very poor | 0.4718 | 0.6040 | Very poor |
| 82 |  |  | 2 | 0.0831 | 3.4317 | Good | 0.0830 | 3.4323 | Good |
| 83 |  |  | 3 | 0.0919 | 3.1726 | Good | 0.0898 | 3.1746 | Good |
| 84 |  |  | 4 | 0.1078 | 2.9214 | Fair | 0.0972 | 2.9310 | Fair |
| 85 |  |  | 5 | 0.1130 | 2.8222 | Fair | 0.1009 | 2.8229 | Fair |
| 86 |  |  | 6 | 0.1213 | 2.7676 | Fair | 0.1029 | 2.7685 | Fair |
| 87 |  |  | 7 | 0.1255 | 2.7375 | Fair | 0.1039 | 2.7428 | Fair |
| 88 |  |  | 8 | 0.1295 | 2.7383 | Fair | 0.1039 | 2.7431 | Fair |
| 89 |  |  | 9 | 0.1337 | 2.7217 | Fair | 0.1044 | 2.7290 | Fair |
| 90 |  |  | 10 | 0.1396 | 2.6970 | Fair | 0.1056 | 2.6988 | Fair |
| 91 | SG(9) | iPLS | 1 | 0.1771 | 1.6091 | Very poor | 0.1770 | 1.6099 | Very poor |
| 92 |  |  | 2 | 0.1495 | 1.9060 | Very poor | 0.1495 | 1.9061 | Very poor |
| 93 |  |  | 3 | 0.0861 | 3.3103 | Good | 0.0861 | 3.3112 | Good |
| 94 |  |  | 4 | 0.0878 | 3.2468 | Good | 0.0877 | 3.2474 | Good |
| 95 |  |  | 5 | 0.0881 | 3.2346 | Good | 0.0880 | 3.2367 | Good |
| 96 |  |  | 6 | 0.0882 | 3.2292 | Good | 0.0882 | 3.2298 | Good |
| 97 |  |  | 7 | 0.0877 | 3.2576 | Good | 0.0874 | 3.2589 | Good |
| 98 |  |  | 8 | 0.0877 | 3.2273 | Good | 0.0882 | 3.2307 | Good |
| 99 |  |  | 9 | 0.0863 | 3.1830 | Good | 0.0895 | 3.1845 | Good |
| 100 |  |  | 10 | 0.0886 | 3.1001 | Good | 0.0918 | 3.1027 | Good |
| 101 | SG(9) | BiPLS | 1 | 0.1771 | 1.6091 | Very poor | 0.1770 | 1.6099 | Very poor |
| 102 |  |  | 2 | 0.1495 | 1.9060 | Very poor | 0.1495 | 1.9061 | Very poor |
| 103 |  |  | 3 | 0.0861 | 3.3103 | Good | 0.0861 | 3.3112 | Good |
| 104 |  |  | 4 | 0.0878 | 3.2468 | Good | 0.0877 | 3.2474 | Good |
| 105 |  |  | 5 | 0.0881 | 3.2346 | Good | 0.0880 | 3.2367 | Good |
| 106 |  |  | 6 | 0.0882 | 3.2292 | Good | 0.0882 | 3.2298 | Good |
| 107 |  |  | 7 | 0.0877 | 3.2576 | Good | 0.0874 | 3.2589 | Good |
| 108 |  |  | 8 | 0.0877 | 3.2273 | Good | 0.0882 | 3.2307 | Good |
| 109 |  |  | 9 | 0.0863 | 3.1830 | Good | 0.0895 | 3.1845 | Good |
| 110 |  |  | 10 | 0.0886 | 3.1001 | Good | 0.0918 | 3.1027 | Good |
| 111 | SG(9) | SiPLS | 1 | 0.3233 | 0.8818 | Very poor | 0.3231 | 0.8820 | Very poor |
| 112 |  |  | 2 | 0.1213 | 2.3494 | Poor | 0.1212 | 2.3505 | Poor |
| 113 |  |  | 3 | 0.0909 | 3.1359 | Good | 0.0908 | 3.1380 | Good |
| 114 |  |  | 4 | 0.0859 | 3.3182 | Good | 0.0858 | 3.3195 | Good |
| 115 |  |  | 5 | 0.0747 | 3.8123 | Very good | 0.0746 | 3.8212 | Very good |
| 116 |  |  | 6 | 0.0728 | 3.9096 | Very good | 0.0728 | 3.9166 | Very good |
| 117 |  |  | 7 | 0.0741 | 3.8710 | Very good | 0.0736 | 3.8731 | Very good |
| 118 |  |  | 8 | 0.0741 | 3.8223 | Very good | 0.0745 | 3.8230 | Very good |
| 119 |  |  | 9 | 0.0757 | 3.7592 | Very good | 0.0757 | 3.7633 | Very good |
| 120 |  |  | 10 | 0.0784 | 3.6839 | Very good | 0.0773 | 3.6854 | Very good |

***The unit of RMSEP is mg/mL.**
