# Supplementary material for: Pharmaceutical Analysis Model Robustness From Bagging-PLS and PLS Using Systematic Tracking Mapping
Source: Front Chem. 2018 Jul 6;6:262. doi: 10.3389/fchem.2018.00262 (PMC6043861; doi:10.3389/fchem.2018.00262)
Supplement: Supplementary file 3 [file Table_3.DOC]

**Supplementary Table S3. The parameters of PLS and Bagging-PLS models of tablet and *Lonicera japonica* via** different spectra pretreatment and variables selections.

| Analytes | Pretreamt | Variable selection | Latent factor | PLS | | | Bagging-PLS | | |
| --- | --- | --- | --- | --- | --- | --- | --- | --- | --- |
|  |  |  |  | RMSEP | RPD | Classification | RMSEP | RPD | Classification |
| Tablet | Raw | iPLS | 3 | 0.5165 | 2.5742 | Fair | 0.5164 | 2.5755 | Fair |
|  |  | BiPLS | 4 | 0.5429 | 2.4489 | Poor | 0.5426 | 2.4511 | Poor |
|  |  | SiPLS | 3 | 0.5507 | 2.4162 | Poor | 0.5504 | 2.4265 | Poor |
|  | 1st | iPLS | 2 | 1.4925 | 0.8958 | Very Poor | 1.4828 | 0.8969 | Very Poor |
|  |  | BiPLS | 2 | 1.4925 | 0.8958 | Very Poor | 1.4828 | 0.8969 | Very Poor |
|  |  | SiPLS | 2 | 1.4917 | 0.8916 | Very Poor | 0.4915 | 0.8917 | Very Poor |
|  | 2nd | iPLS | 2 | 1.4756 | 0.9018 | Very Poor | 1.4745 | 0.9020 | Very Poor |
|  |  | BiPLS | 2 | 1.4756 | 0.9018 | Very Poor | 1.4745 | 0.9020 | Very Poor |
|  |  | SiPLS | 2 | 1.4760 | 0.9062 | Very Poor | 1.4758 | 0.9073 | Very Poor |
|  | SG(9) | iPLS | 3 | 0.5178 | 2.5687 | Fair | 0.5177 | 2.5690 | Fair |
|  |  | BiPLS | 4 | 0.5485 | 2.4254 | Poor | 0.5481 | 2.4266 | Poor |
|  |  | SiPLS | 4 | 0.5498 | 2.4540 | Poor | 0.5417 | 2.4551 | Poor |
| *Lonicera japonica* | Raw | iPLS | 2 | 0.0893 | 3.1917 | Good | 0.0891 | 3.1966 | Good |
|  |  | BiPLS | 2 | 0.0893 | 3.1917 | Good | 0.0891 | 3.1966 | Good |
|  |  | SiPLS | 2 | 0.1214 | 2.3479 | Poor | 0.1213 | 2.3495 | Poor |
|  | 1st | iPLS | 1 | 0.1945 | 1.4665 | Very Poor | 0.1942 | 1.4670 | Very Poor |
|  |  | BiPLS | 1 | 0.2969 | 0.9569 | Very Poor | 0.2965 | 0.9611 | Very Poor |
|  |  | SiPLS | 1 | 0.2607 | 1.0926 | Very Poor | 0.2606 | 1.0934 | Very Poor |
|  | 2nd | iPLS | 1 | 0.9160 | 0.3109 | Very Poor | 0.9144 | 0.3116 | Very Poor |
|  |  | BiPLS | 1 | 0.7919 | 0.3594 | Very Poor | 0.7908 | 0.3603 | Very Poor |
|  |  | SiPLS | 1 | 0.4725 | 0.6035 | Very Poor | 0.4718 | 0.6040 | Very Poor |
|  | SG(9) | iPLS | 2 | 0.1495 | 1.9060 | Very Poor | 0.1495 | 1.9061 | Very Poor |
|  |  | BiPLS | 2 | 0.1495 | 1.9060 | Very Poor | 0.1495 | 1.9061 | Very Poor |
|  |  | SiPLS | 2 | 0.1213 | 2.3494 | Poor | 0.1212 | 2.3505 | Poor |
